# Association of LEPTIN and other inflammatory markers with preeclampsia: A systematic review

**DOI:** 10.3389/fphar.2022.966400

**Published:** 2022-08-10

**Authors:** Eduardo Carvalho de Arruda Veiga, Henri Augusto Korkes, Karina Bezerra Salomão, Ricardo Carvalho Cavalli

**Affiliations:** ^1^ Department of Obstetrics and Gynecology, University Hospital, Ribeirão Preto Medical School, University of São Paulo, São Paulo, Brazil; ^2^ Department of Pediatrics, Ribeirão Preto Medical School University of São Paulo, Ribeirão Preto, Brazil

**Keywords:** preeclaimpsia, inflammatory, markers, C reaction protein, HDL

## Abstract

**Background:** Preeclampsia is a serious pregnancy complication that affects 5%–10% of the obstetric population.

**Objective:** To study inflammatory markers associated with preeclampsia.

**Search Strategy:** Searches of articles on the topic published over a 10-year period (2009–2019) were performed in three databases (PubMed, Cochrane, and Embase) using the keywords preeclampsia and inflammatory markers. The PubMed search using 10 years and humans as filters retrieved 124 articles. Using an advanced search strategy, 0 articles were identified in Embase and 10 articles in Cochrane. After screening and eligibility assessment, 13 articles were included in the systematic review and meta-analysis. Meta-analysis and quality assessment of the studies were performed using the Review Manager 5.3 program.

**Results:** For meta-analysis, women with preeclampsia were compared to control women, i.e., pregnancies without arterial hypertension. Leptin levels were significantly higher (*p* < 0.0002) in women with preeclampsia compared to controls. Total cholesterol was also significantly elevated in women with preeclampsia (*p* < 0.0001). There was no significant difference in HDL between groups, but women with preeclampsia had significantly increased LDL (*p* < 0.01). The same was observed for triglycerides, which were significantly increased in women with preeclampsia (*p* < 0.04) compared to controls. Analysis of TNF-alpha, an important inflammatory marker, showed higher levels in women with preeclampsia (*p* < 0.03) compared to controls. The same was observed for another important inflammatory marker, interleukin 6, which was significantly increased in women with preeclampsia (*p* < 0.0002). There was a significant increase of C-reactive protein in women with preeclampsia (*p* < 0.003) compared to controls.

**Conclusion:** Women with preeclampsia have increased levels of inflammatory markers compared to control women.

## Introduction

Hypertensive disorders are one of the most common complications of pregnancy worldwide and account for about 20% of deaths of pregnant women in Latin America according to data from a study by the World Health Organization published in 2014. These disorders are serious conditions whose prevalence ranges from 3% to 9% (Brown et al., 2018). Among hypertensive disorders, preeclampsia is a matter of concern because of its impact on maternal and neonatal health. Preeclampsia is a leading cause of maternal and perinatal morbidity and mortality, affecting approximately 5% of all pregnancies worldwide (Jeyabalan, 2013; Brown et al., 2018; Wang et al., 2021).

Preeclampsia is a disorder of pregnant women that occurs after 20 weeks of gestation, although it can present as late as 4–6 weeks postpartum. The clinical manifestations include hypertension and proteinuria in 24 h > 0.3 g/L, with or without edema, but the disease may even affect all organ systems (Rana et al., 2019).

Increased maternal inflammatory status and oxidative stress associated with excess adipose tissue are considered the main biological triggers of abnormal early placentation among obese subjects. Placental defects can lead to increased resistance in the maternal-fetal circulation, triggering the development of preeclampsia. (Mayrink et al., 2018).

Adiponectin and leptin are adipokines, hormones produced mainly by adipose tissue, that are responsible for the regulation of lipid metabolism, placental angiogenesis, insulin sensitivity, inflammatory processes, and trophoblast invasion (Thagaard et al., 2019a; Kaze et al., 2021; Bhat et al., 2022). Adipokines appear to be involved in the complex mechanisms of early pregnancy and implantation and may therefore play a potential role in the development of preeclampsia. In early pregnancy, maternal leptin concentration correlates strongly with pre-pregnancy body mass index. Subsequently, placental production of leptin contributes to the increase in maternal leptin concentration during pregnancy (Thagaard et al., 2019a; Kaze et al., 2021; Bhat et al., 2022). Beneventi et al., 2020 investigated maternal and fetal plasma levels of leptin in pregnancies complicated by obesity and preeclampsia. The authors found that pregnant women with obesity had higher serum leptin levels than normal-weight subjects with and without hypertension or normotensive subjects with obesity (Larsen et al., 2019; Beneventi et al., 2020; Abraham and Romani, 2022).

From a practical and clinical point of view, maternal serum adiponectin and leptin can be used as markers to identify women with a predisposition to developing hypertension during pregnancy and thus can permit early detection. The importance of further studies on these adipokines lies in the fact that these proteins may be useful in the future not only as metabolic predictors but also for the prevention of arterial hypertension, diabetes, and diabetes. Atherogenesis as has been shown experimentally (Farkhondeh et al., 2020; Kim et al., 2022).

The objective of this study was to investigate inflammatory markers such as adiponectin and leptin associated with preeclampsia.

## Methods

**FIGURE 1:**
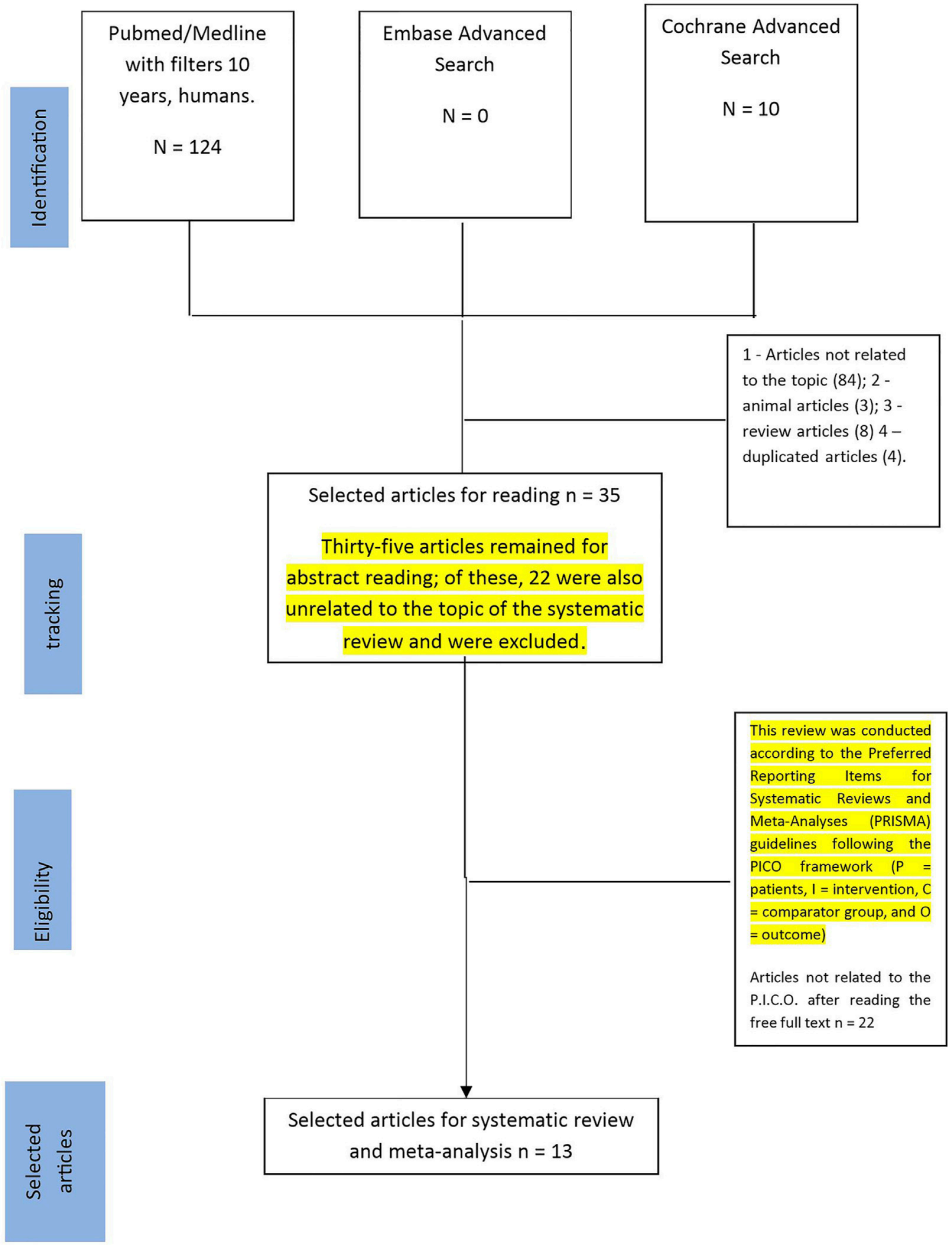
Flowchart for selection of studies.

The search strategy followed the recommendations of Berstock et al., 2019 (Berstock and Whitehouse, 2019). Articles published from January 2009 to November 2019 in PubMed, Embase, and Cochrane were eligible. First, we selected keywords from related articles. Medical Subject Headings (MeSH) were then used to find more related keywords with similar meanings: (“inflammatory markers” [MeSH Terms] OR (“preeclampsia” [All Fields] AND (“inflammatory markers and preeclampsia”) [MeSH Terms] [All Fields]. Searches were performed in the three databases. PubMed searches using 10 years and humans as filters resulted in 124 potential articles. Searching only the title in Embase resulted in 0 articles and the Cochrane database search retrieved 10 articles, all involving humans. After a first selection, 84 articles were not selected because they were unrelated to the topic of the systematic review, 3 articles involved animals, 4 articles were reviews, and 4 articles were duplicates. Thirty-five articles remained for abstract reading; of these, 22 were also unrelated to the topic of the systematic review and were excluded. Finally, 13 articles were included in the systematic review and meta-analysis (Figure 1). This review was conducted according to the Preferred Reporting Items for Systematic Reviews and Meta-Analyses (PRISMA) guidelines following the PICO framework (P = patients, I = intervention, C = comparator group, and O = outcome) (Eriksen and Frandsen, 2018).

Two researchers with experience in compelling systematic reviews (E.C.V. and _KBS_) independently and blindly retrieved the articles and evaluated the titles and abstracts following the inclusion and exclusion criteria according to the PICO components (Eriksen and Frandsen, 2018). The selected articles were then critically evaluated for inclusion in the review or exclusion. Disagreements regarding the selection of studies were resolved by a third reviewer (HK).

The following data were extracted from the studies selected for the systematic review and entered into a table: author names and year of publication, study design, the definition of preeclampsia (systolic blood pressure, diastolic blood pressure, and/or diabetes), number of participants in the study, maternal age (years), inflammatory markers study and outcome (Table 1). The RevMan version 5.3 program (Cochrane Collaboration, Oxford, United Kingdom) was used for meta-analysis. A random-effect model was used to estimate heterogeneity.

**TABLE 1:** Characteristics of studies on women with preeclampsia.

Author, publication year	Country	Study design	Definition of PE: SBP/DBP or diabetes	No of participants in the study	Maternal age (years)	Inflammatory markers studied	Outcome
Valencia-Ortega et al., 2018	Mexico	Cross-sectional study	142/87 mmHg	50 PE/50 control	28.5 PE/28 control	TNF-α, IL-6, IL-8, IL-10, IL-1RA, ICAM-1, VCAM-1	PE is associated with a pro-inflammatory placental state
Mouse et al., 2017	Australia	Randomized controlled trial	Overweight type 1 or 2 diabetes	102	31.9	Triglycerides, total cholesterol, HDL, LDL, vitamin D, adiponectin, IL-6, MCP1	Vitamin D in obese pregnant women is associated with an increase in cardiovascular risk during pregnancy and this association is mediated by adiponectin
Kharb et al., 2017	India	Cross-sectional study	140/90 mmHg	50	—	Triglycerides, total cholesterol, HDL, LDL, leptin, IGF-1	Alterations in biochemical markers of growth and obesity occur in mothers and fetuses and modifications in the uterine environment can contribute to prevent future cardiovascular risk
Perichart-Pereira et al., 2017	Mexico	Prospective cohort	140/90 mmHg	177	27	Insulin, total cholesterol, HDL, LDL, triglycerides, IL-1β, leptin, adiponectin	Maternal weight status affected the concentrations of insulin, leptin, adiponectin, triglycerides and C-reactive protein throughout pregnancy
Gauster et al., 2017	Austria	Cross-sectional study	diabetes	17	31	TNF-α, HSP70, HO1	Diabetes increases placental cellular stress in the first trimester
Bashir et al., 2017	Saudi Arabia/Egypt	Cross-sectional study	140/90 mmHg	158	27	Leptin, TNF-α, SOD, NO, IL-6	The combination of PE and high altitude residence resulted in significantly elevated maternal serum leptin
Ferguson et al., 2016	United States	Prospective birth cohort	140/90 mmHg	441	20–40	C-reactive protein, IL-1β, IL-6, IL-10, TNF-α	Demonstration of significant associations between biomarkers of inflammation and oxidative stress and PE
Estensen et al., 2015	Norway	Longitudinal study	140/90 mmHg	95	32	STNFR1, sVCAM.	Preeclamptic pregnancies are characterized by increased circulating levels of systemic and vascular inflammatory markers
Udenze et al., 2015	Nigeria	Case-control study	160/110 mmHg	100	32	IL-6, CRP, TNF-α	The inflammatory cytokines IL-6, TNF-α and C-reactive protein are elevated in severe PE
Drost et al., 2014	The Netherlands	Retrospective cohort	130/90 mmHg	671	39	Adiponectin, Leptin, sVCAM.	The authors demonstrated an independent association of preeclampsia with SE-selectin and PAPPA, which may contribute to future cardiovascular events in women post-PE
Du et al., 2013	United States	Cross-sectional study	PE with diabetes	66	30	C-reactive protein, IL-1ra	In pregnant women with diabetes, elevated C-reactive protein and IL-1ra were associated with subsequent PE
Babu et al., 2012	India	Case-control study	140/90 mmHg	90	23	C-reactive protein	Oxidative stress and the inflammatory response are greater in women with PE compared to pregnant women with gestational hypertension
Can et al., 2011	Turkey	Cross-sectional study	140/90 mmHg	104	30	C-reactive protein	The results confirm that inflammatory reactions are closely associated with PE

PE, preeclampsia; SBP, systolic blood pressure; DBP, diastolic blood pressure.

## Statistical analysis

In the statistics of the preeclampsia groups, the means, standard deviations and the total number of women were described, and in the control groups also each author. And also the mean difference, randomized with a confidence interval of 95%.Meta-analysis was carried out with the Review Manager 5.3 software program (Cochrane Collaboration, Oxford, United Kingdom) by comparing the means and standard deviations of the preeclampsia and the control groups. The random-effects model was used in the case of heterogeneity (DerSimonian and Kacker, 2007; von Hippel, 2015).

## Results

**FIGURE 2:**
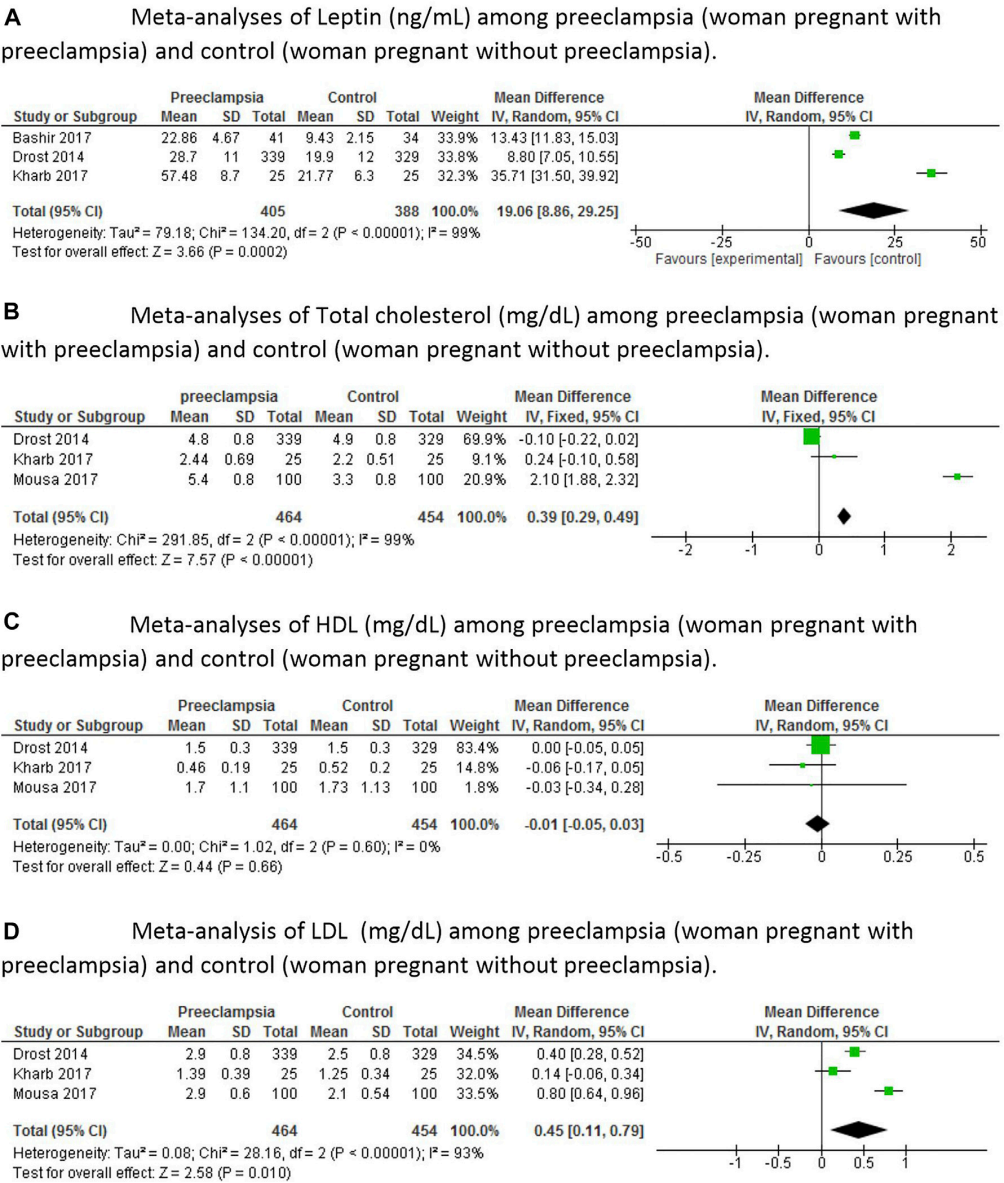
Meta-analyses of inflammatory markers among pregnant women with preeclampsia and control pregnant women (control), that is, without preeclampsia. **(A)**—Meta-analyses of Leptin (ng/ml) among preeclampsia (woman pregnant with preeclampsia) and control (woman pregnant without preeclampsia). **(B)**—Meta-analyses of Total cholesterol (mg/dl) among preeclampsia (woman pregnant with preeclampsia) and control (woman pregnant without preeclampsia). **(C)**—Meta-analyses of HDL (mg/dl) among preeclampsia (woman pregnant with preeclampsia) and control (woman pregnant without preeclampsia). **(D)**—Meta-analysis of LDL (mg/dl) among preeclampsia (woman pregnant with preeclampsia) and control (woman pregnant without preeclampsia).

**FIGURE 3:**
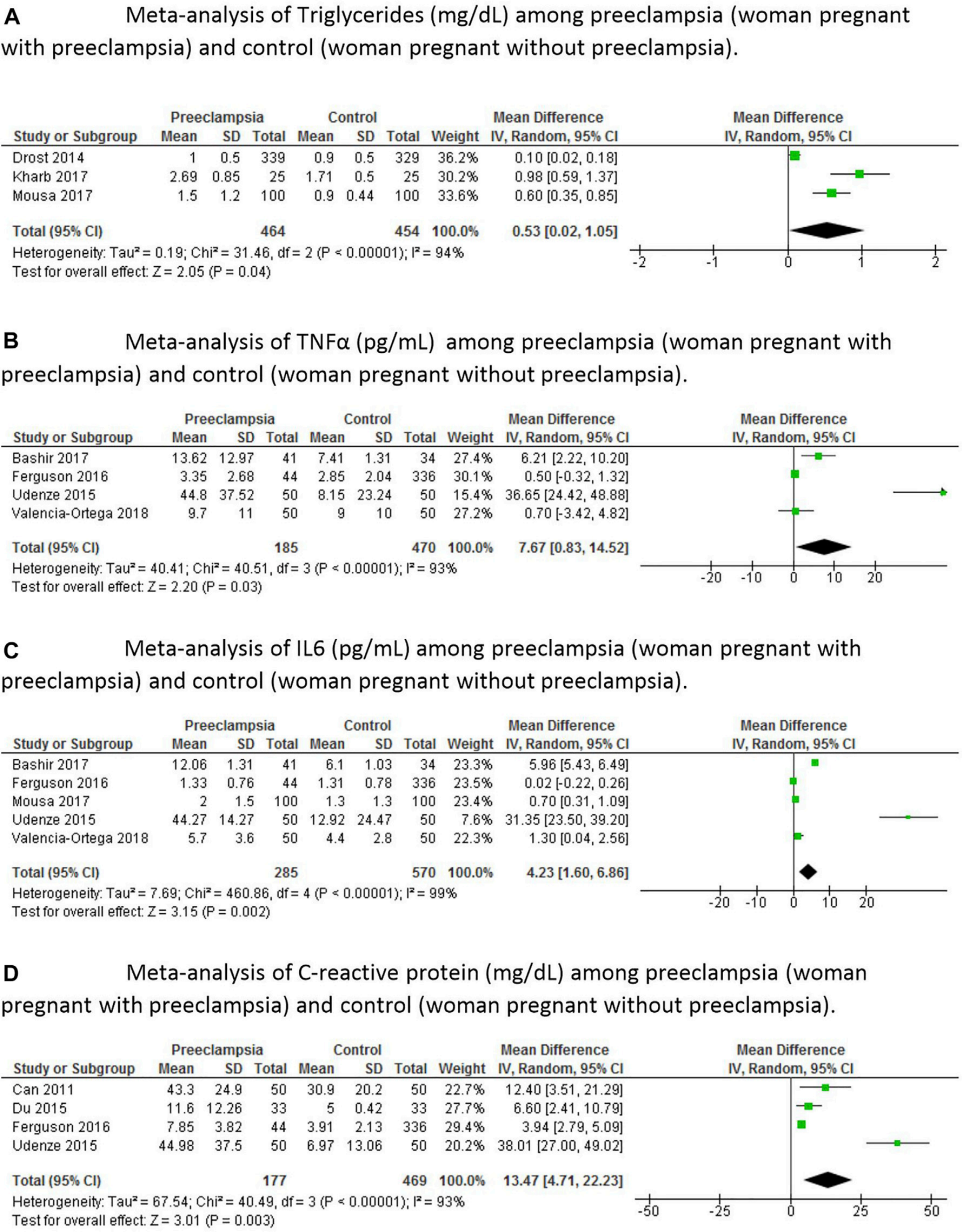
Meta-analyses of inflammatory markers among pregnant women with preeclampsia and control pregnant women (control), that is, without preeclampsia. **(A)**—Meta-analysis of Triglycerides (mg/dl) among preeclampsia (woman pregnant with preeclampsia) and control (woman pregnant without preeclampsia). **(B)**—Meta-analysis of TNFα (pg/ml) among preeclampsia (woman pregnant with preeclampsia) and control (woman pregnant without preeclampsia). **(C)**—Meta-analysis of IL6 (pg/ml) among preeclampsia (woman pregnant with preeclampsia) and control (woman pregnant without preeclampsia). **(D)**—Meta-analysis of C-reactive protein (mg/dl) among preeclampsia (woman pregnant with preeclampsia) and control (woman pregnant without preeclampsia).

In the meta-analyses, we first evaluated leptin whose levels were significantly increased (*p* < 0.0002) in the group of women with preeclampsia compared to the control group (Figure 2A). Total cholesterol was also elevated in the group with preeclampsia (*p* < 0.0001) compared to the control group (Figure 2B). There was no difference in HDL between groups (*p* = 0.66), probably because of the high standard deviation in one of the articles analyzed (Figure 2C); however, LDL was significantly increased in women with preeclampsia (*p* < 0.01) compared to control (Figure 2D). There was also a difference in triglycerides (*p* < 0.04) between the experimental group and the control group (Figure 3A). When we analyzed other inflammatory markers such as tumor necrosis factor alpha (TNF-α), we observed significantly increased levels of this marker in the group of women with preeclampsia (*p* < 0.03) compared to women with normal pregnancies (Figure 3B). The same was observed for interleukin 6 (IL-6) (Figure 3C), with this marker being significantly increased in the group of women with preeclampsia (*p* < 0.002). There was a significant increase of C-reactive protein in women with preeclampsia (*p* < 0.003) compared to pregnant women without hypertension (Figure 3D).

**FIGURE 4:**
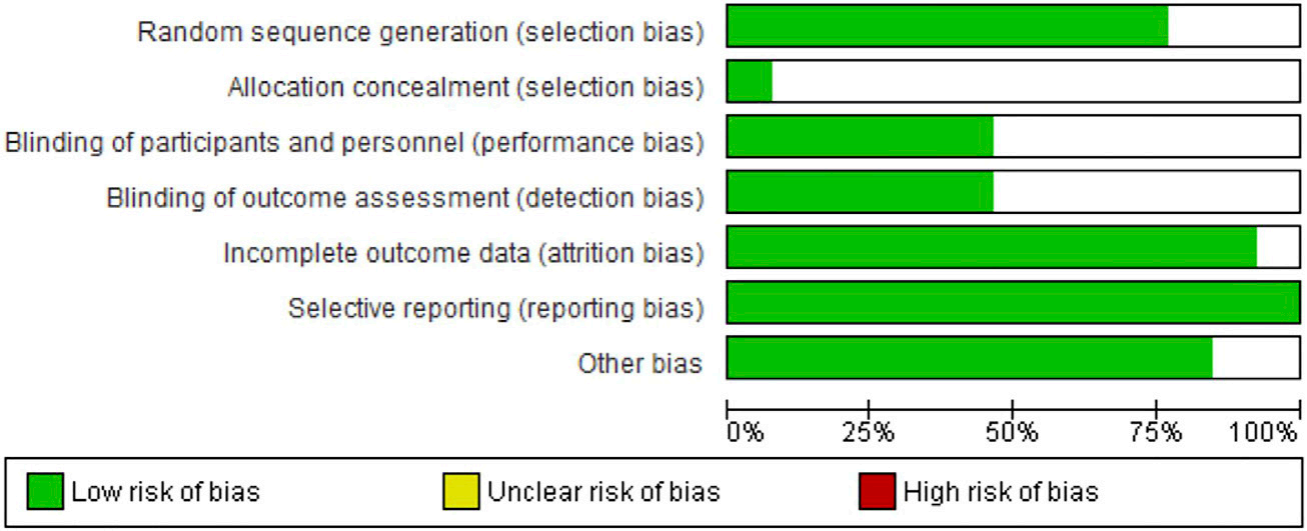
Risk of bias graph: review authors’ judgements about each risk of bias item presented as percentages across all included studies.

**FIGURE 5:**
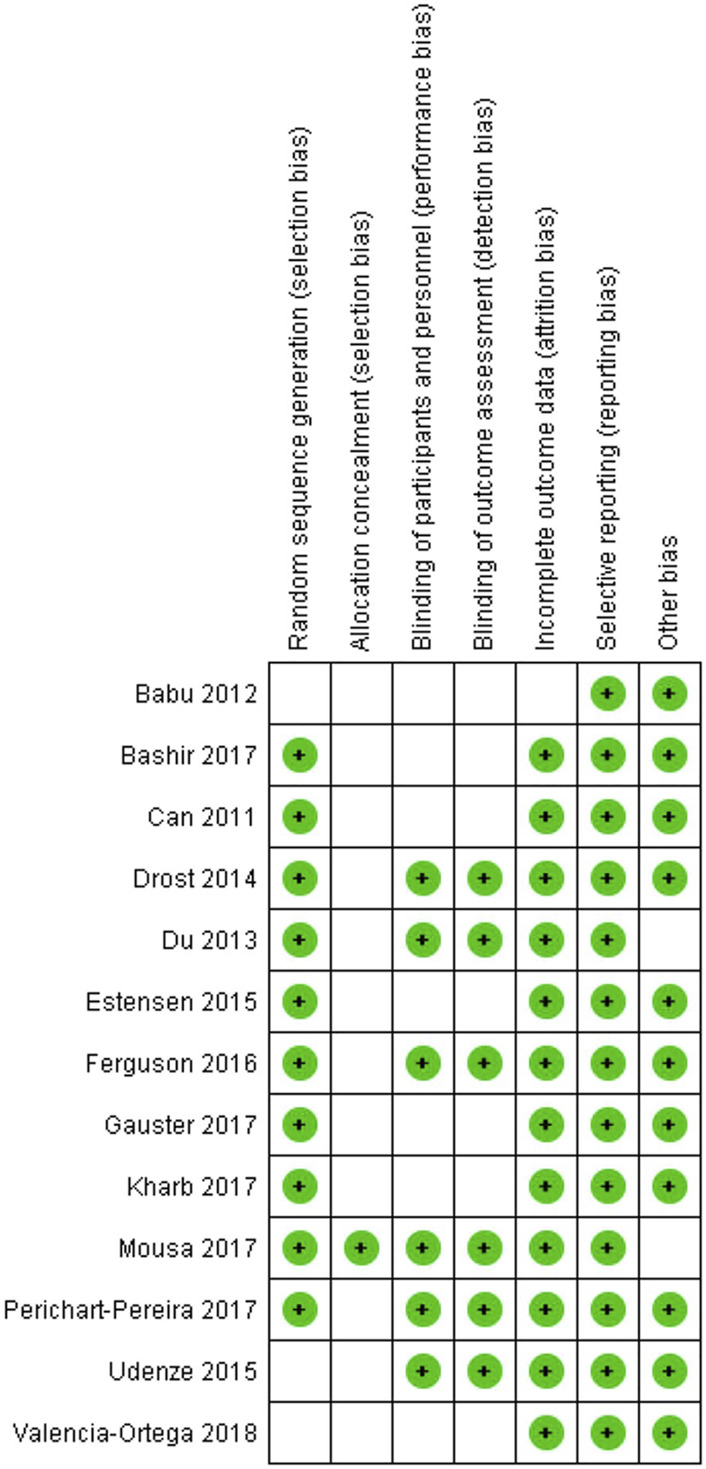
Risk of bias summary: review authors’ judgements about each risk of bias item for each included study.

The following results of the risk of bias assessment should be highlighted. All articles were classified as low risk of bias for selective reporting. When we analyzed incomplete outcome data and other bias in the 13 articles, 12 were rated as low risk of bias and only one as unclear risk of bias (Figure 4). Regarding random sequence generation, most articles (10 articles or 76.92%) were at low risk of bias and only 3 articles were at unclear risk of bias. When we analyzed blinding of participants and personnel and blinding of outcome assessment, slightly less than half of the articles (6 articles) were rated as low risk of bias and the remaining 7 articles as unclear risk of bias. Most articles had an unclear risk of bias only for allocation concealment, with only one article showing low risk of bias (Figure 5).

## Discussion

The main finding of this study was that women with preeclampsia had increased levels of inflammatory markers compared to pregnant women without this condition. The inflammatory markers showing a significant difference were leptin, TNF-α, IL-6, and C-reactive protein.

Adiponectin is an anti-inflammatory factor, that is, associated with the physiopathology of preeclampsia. Several authors have studied the association between adiponectin and preeclampsia and found conflicting results. Most studies report an increase of adiponectin in the third trimester of gestation in patients with preeclampsia compared to control (Pérez-Pérez et al., 2020; Pheiffer et al., 2021; Lara-Barea et al., 2022), while others found the Other adipokines such as resistin, visfatin and vaspin in gestational diabetes and preeclampsia have been described but their physiological role has yet to be established and work has been more descriptive regarding these other adipokines (Miehle et al., 2012). In pregnant women plasma levels of resistin are elevated compared to non-pregnant women, however in gestational diabetes there are inconsistent data in the literature with some studies demonstrating elevation of resistin while others found decreased levels. Visfatin has anti-apoptotic properties and recombinant human visfatin treatment of human fetal membranes causes a significant increase in inflammatory cytokines, but its role in preeclampsia and gestational diabetes remains contradictory. In the case of vaspin, serum levels are not associated with markers of insulin resistance in pregnant patients. Recent studies associate resistin and visfatin as predictors of gestational diabetes mellitus and also that these adipolines are found in tissues such as adipose, subcutaneous adipose, placenta and cord blood (Bawah et al., 2019; Valencia-Ortega et al., 2022). Meta-analysis of the adiponectin results was not possible in our study because the selected articles did not report these results.

Leptin is an adipokine expressed in adipose tissue, that is, involved in energy expenditure and the modulation of insulin resistance (Thagaard et al., 2019b). This hormone is also produced by trophoblastic cells of the placenta (Beneventi et al., 2020). A recent study suggests that high preconceptual leptin levels may be a body mass index-independent risk factor for gestational diabetes mellitus and also a body mass index-dependent risk factor for hypertensive pregnant women (Peltokorpi et al., 2022).

Some studies measuring leptin in the second and third trimesters of gestation found an increase in leptin levels in pregnant women with preeclampsia compared to the control group (Thagaard et al., 2019b; de Knegt et al., 2021). Our meta-analysis supports these results since leptin was also found to be increased in patients with preeclampsia. Another study suggested that leptin may be a predictor in the obese population and elevated leptin levels have also been associated with cardiovascular disease (Nzelu et al., 2020; Stefańska et al., 2021).

The systematic review of Black et al., 2018 (Black and Horowitz, 2018) examined 73 studies published between 1998 and 2016 and revealed that some inflammatory markers such as IL-6, IL-8, TNF-α, and C-reactive protein may be useful for identifying women at risk of developing preeclampsia, in agreement with the results of our meta-analysis. Likewise, two other systematic reviews (30,31) also identified elevated levels of TNF-α, IL-6, and IL-10 in studies on preeclampsia.

With respect to IL-6, studies have demonstrated a significant increase of this cytokine in pregnant women with preeclampsia complications when compared to pregnant controls (Nzelu et al., 2020; Stefańska et al., 2021). Another studied used inflammatory marker is TNF-α. The results are contradictory, with most studies reporting an increase of this marker in preeclampsia, including the present study in which meta-analysis showed a significant difference in TNF-α levels compared to control. On the other hand, some studies did not find a significant difference between pregnant women with preeclampsia and pregnant controls (Lau et al., 2013; Black and Horowitz, 2018).

C-reactive protein is an acute-phase protein of inflammation and is frequently studied as a marker of preeclampsia. Our results also showed an increase in this protein, in agreement with the literature. In one systematic review, most studies (18 studies) showed elevated levels of C-reactive protein in women with preeclampsia compared to the control groups, while a minority of 10 studies found that C-reactive protein levels did not differ from controls (Black and Horowitz, 2018).

Abnormal lipid profiles have also been linked to both preeclampsia and impaired metabolic health in obese people. High levels of LDL have been observed in patients with preeclampsia in the third trimester of gestation compared to normotensive controls (Alahakoon et al., 2020). Our results revealed no significant difference in LDL between the preeclampsia and control groups. There was also no difference in HDL between the groups analyzed. Other studies found low HDL levels and high triglyceride levels in patients with preeclampsia when compared to controls (Dong et al., 2021; Zhou et al., 2021). In our study, triglyceride levels were higher in patients with preeclampsia than in the control group. Furthermore, high triglyceride and LDL levels and low HDL levels have been associated with impaired cardiovascular health (Soppert et al., 2020).

Future perspectives for this topic should include more clinical and experimental research on inflammatory markers in preeclampsia and gestational diabetes mellitus with a substantial number of women studied and further research is needed on these more common cardiovascular markers and others less common cardiovascular markers.

## Conclusion

We concluded that some inflammatory markers in our meta-analysis such as leptin, total cholesterol, triglycerides, TNFα and C-reactive protein were increased in pregnant women with preeclampsia compared to pregnant control women, so inflammatory markers are important markers of preeclampsia.

## Data Availability

The original contributions presented in the study are included in the article/supplementary materials, further inquiries can be directed to the corresponding author.
